# Draft Genome Sequence of a Basidiomycetous Yeast, Ustilago shanxiensis CBS 10075, Which Produces Mannosylerythritol Lipids

**DOI:** 10.1128/MRA.00706-21

**Published:** 2021-12-02

**Authors:** Keisuke Wada, Hideaki Koike, Tomotake Morita

**Affiliations:** a Research Institute for Sustainable Chemistry, National Institute of Advanced Industrial Science and Technology, Tsukuba, Ibaraki, Japan; b Bioproduction Research Institute, National Institute of Advanced Industrial Science and Technology, Tsukuba, Ibaraki, Japan; University of California, Riverside

## Abstract

The basidiomycetous yeast Ustilago shanxiensis CBS 10075, which was isolated from a wilting leaf in China, produces mannosylerythritol lipid (MEL) biosurfactants. Here, we report the draft genome sequence of U. shanxiensis CBS 10075, which was 21.7 Mbp in size, with a GC content of 52.55%, comprising 65 scaffolds.

## ANNOUNCEMENT

Ustilago shanxiensis CBS 10075 (renamed from Pseudozyma shanxiensis CBS 10075) is a basidiomycetous yeast that was isolated from a withered leaf of Quercus mongolica Fisch in China (1). Yeast strains of the genus *Pseudozyma* typically produce functional glycolipids, such as mannosylerythritol lipids (MELs). U. shanxiensis CBS 10075 produces MEL-C compounds as a mixture of 4-*O*-[(2′,4′-di-*O*-acetyl-3′-*O*-alka(e)noyl)-β-d-mannopyranosyl]-d-erythritol and 4-*O*-[(4′-*O*-acetyl-3′-*O*-alka(e)noyl-2′-*O*-butanoyl)-β-d-mannopyranosyl]-d-erythritol. The MELs produced by U. shanxiensis CBS 10075 are more hydrophilic than those typically produced by yeasts such as Moesziomyces antarcticus (renamed from Pseudozyma antarctica) (2).

U. shanxiensis CBS 10075 was grown at 25°C for 48 h in 30 ml of YM medium at 250 rpm. The total genomic DNA was obtained by phenol-chloroform extraction followed by isopropanol precipitation. The paired-end DNA library (insert size, ∼500 bp) of the U. shanxiensis genome was prepared using a NEBNext Ultra DNA library preparation kit for Illumina (New England BioLabs, Ipswich, MA, USA), and sequencing was performed using the MiSeq platform (Illumina, San Diego, CA, USA). Sequencing data comprising a total of 10,427,528 paired-end reads were generated; each read was 250 bp in length. A mate-paired library (insert size, ∼3,200 bp) was then prepared using a Nextera mate pair sample preparation kit (Illumina), generating 9,469,570 mate-paired reads. The quality of paired-end and mate-paired reads was checked by FastQC version 0.11.2. Genome sequence assembly using the ALLPATHS-LG version R46449 assembler (3) provided 65 scaffolds (*N*_50_, 770,995 bp; scaffold *L*_50_, 8) composed of 271 contigs generated from the paired-end and mate-paired reads, with 81× and 64.5× sequence coverage, respectively. The U. shanxiensis CBS 10075 draft genome size was 21.7 Mbp (GC content, 52.55%). The length of the longest scaffold was 2,733 kbp. Five MEL biosynthesis genes were predicted using AUGUSTUS version 2.5.5 (4) and annotated using NCBI BLAST version 2.2.29 with RefSeq version 65 (5, 6). Default parameters were used except where otherwise noted.

The CBS 10075 genome sequence contained a conserved MEL biosynthesis gene cluster comprising five genes in scaffold 6, namely, *UshEMT1* (an erythritol-mannosyl-transferase), *UshMAC1* and *UshMAC2* (acyl-coenzyme A [CoA]-dependent acyltransferases), *UshMAT1* (an acetyltransferase), and *UshMMF1* (a putative MEL transporter). This cluster of MEL biosynthesis genes was first reported in Ustilago maydis (7), and the evolutionary relationships among other MEL-producing organisms has since been investigated by comparing the amino acid sequences of enzymes in the MEL biosynthetic pathway (8). Recently, Ustilago hordei UM4857-4 was found to possess a cluster of MEL biosynthesis genes (9) that showed the greatest identity to U. shanxiensis CBS 10075 in this study (*UshEMT1*, 87.7%; *UshMAC1*, 80.8%; *UshMAC2*, 87.3%; *UshMAT1*, 57.0%; *UshMMF1*, 87.1%). The genome sequence of U. shanxiensis CBS 10075 will improve our understanding of the molecular mechanisms driving MEL production and will enable the development of MELs tailored for a range of industries.

### Data availability.

The nucleic acid sequence of the U. shanxiensis CBS 10075 genome has been deposited in DDBJ/EMBL/GenBank under accession numbers BPMX01000001 to BPMX01000065. The DDBJ Sequence Read Archive (DRA) accession number is DRR306489. The protein identification numbers are as follows: *UshMAC2*, GIZ99647; *Ush*EMT1, GIZ99648; *Ush*MAC1, GIZ99649; *Ush*MMF1, GIZ99650; and *Ush*MAT1, GIZ99651.

## References

[1] Wang Q-M, Jia J-H, Bai F-Y. 2006. *Pseudozyma hubeiensis* sp. nov. and *Pseudozyma shanxiensis* sp. nov., novel ustilaginomycetous anamorphic yeast species from plant leaves. Int J Syst Evol Microbiol 56:289–293. doi:10.1099/ijs.0.63827-0. 16403900

[2] Fukuoka T, Morita T, Konishi M, Imura T, Kitamoto D. 2007. Characterization of new types of mannosylerythritol lipids as biosurfactants produced from soybean oil by a basidiomycetous yeast, *Pseudozyma shanxiensis*. J Oleo Sci 56:435–442. doi:10.5650/jos.56.435. 17898510

[3] Gnerre S, Maccallum I, Przybylski D, Ribeiro FJ, Burton JN, Walker BJ, Sharpe T, Hall G, Shea TP, Sykes S, Berlin AM, Aird D, Costello M, Daza R, Williams L, Nicol R, Gnirke A, Nusbaum C, Lander ES, Jaffe DB. 2011. High-quality draft assemblies of mammalian genomes from massively parallel sequence data. Proc Natl Acad Sci USA 108:1513–1518. doi:10.1073/pnas.1017351108. 21187386 PMC3029755

[4] Stanke M, Steinkamp R, Waack S, Morgenstern B. 2004. AUGUSTUS: a Web server for gene finding in eukaryotes. Nucleic Acids Res 32:W309–W312. doi:10.1093/nar/gkh379. 15215400 PMC441517

[5] Altschul SF, Gish W, Miller W, Myers EW, Lipman DJ. 1990. Basic local alignment search tool. J Mol Biol 215:403–410. doi:10.1016/S0022-2836(05)80360-2. 2231712

[6] Tatusova T, Ciufo S, Fedorov B, O’Neill K, Tolstoy I. 2014. RefSeq microbial genomes database: new representation and annotation strategy. Nucleic Acids Res 42:D553–D559. doi:10.1093/nar/gkt1274. 24316578 PMC3965038

[7] Hewald S, Linne U, Scherer M, Marahiel MA, Kämper J, Bölker M. 2006. Identification of a gene cluster for biosynthesis of mannosylerythritol lipids in the basidiomycetous fungus *Ustilago maydis*. Appl Environ Microbiol 72:5469–5477. doi:10.1128/AEM.00506-06. 16885300 PMC1538720

[8] Saika A, Utashima Y, Koike H, Yamamoto S, Kishimoto T, Fukuoka T, Morita T. 2018. Identification of the gene *PtMAT1* encoding acetyltransferase from the diastereomer type of mannosylerythritol lipid-B producer *Pseudozyma tsukubaensis*. J Biosci Bioeng 126:676–681. doi:10.1016/j.jbiosc.2018.05.025. 30037643

[9] Deinzer H-T, Linne U, Xie X, Bölker M, Sandrock B. 2019. Elucidation of substrate specificities of decorating enzymes involved in mannosylerythritol lipid production by cross-species complementation. Fungal Genet Biol 130:91–97. doi:10.1016/j.fgb.2019.05.003. 31103599

